# Cardiac phenotype in *ATP1A3*-related syndromes: A multicenter cohort study

**DOI:** 10.1212/WNL.0000000000010794

**Published:** 2020-11-24

**Authors:** Simona Balestrini, Mohamad A. Mikati, Reyes Álvarez-García-Rovés, Michael Carboni, Arsen S. Hunanyan, Bassil Kherallah, Melissa McLean, Lyndsey Prange, Elisa De Grandis, Alessandra Gagliardi, Livia Pisciotta, Michela Stagnaro, Edvige Veneselli, Jaume Campistol, Carmen Fons, Leticia Pias-Peleteiro, Allison Brashear, Charlotte Miller, Raquel Samões, Vesna Brankovic, Quasar S. Padiath, Ana Potic, Jacek Pilch, Aikaterini Vezyroglou, Ann M.E. Bye, Andrew M. Davis, Monique M. Ryan, Christopher Semsarian, Georgina Hollingsworth, Ingrid E. Scheffer, Tiziana Granata, Nardo Nardocci, Francesca Ragona, Alexis Arzimanoglou, Eleni Panagiotakaki, Inês Carrilho, Claudio Zucca, Jan Novy, Karolina Dzieżyc, Marek Parowicz, Maria Mazurkiewicz-Bełdzińska, Sarah Weckhuysen, Roser Pons, Sergiu Groppa, Daniel S. Sinden, Geoffrey S. Pitt, Andrew Tinker, Michael Ashworth, Zuzanna Michalak, Maria Thom, J. Helen Cross, Rosaria Vavassori, Juan P. Kaski, Sanjay M. Sisodiya

**Affiliations:** From the Department of Clinical and Experimental Epilepsy (S.B., S.M.S.), UCL Queen Square Institute of Neurology, London; Chalfont Centre for Epilepsy (S.B., S.M.S.), Bucks, UK; Division of Pediatric Neurology (M.A.M., A.S.H., B.K., M.M., L.P.), Department of Neurobiology, and Division of Cardiology (M.C.), Department of Pediatrics, Duke University, School of Medicine, Durham, NC; Centre for Inherited Cardiovascular Diseases (R.A.G.-R., J.P.K.), Great Ormond Street Hospital for Children NHS Foundation Trust; Institute of Cardiovascular Science(R.A.G.-R., J.P.K.), University College London, London, UK; Child Neuropsychiatry Unit (E.D.G., A.G., L.P., M.S., E.V.), IRCCs Istituto Giannina Gaslini, Department of Neurosciences, Rehabilitation, Ophthalmology, Genetics and Maternal and Child Health, DINOG-MI, University of Genoa; Department of Pediatric Neuroscience (A.G., T.G., N.N., F.R.), Fondazione IRCCS Istituto Neurologico Carlo Besta; Unit of Child Neuropsychiatry (L.P.), ASST Fatebenefratelli Sacco, Milan, Italy; Paediatric Neurology Department (J.C., C.F., L.P.-P., A.A.), Hospital Sant Joan de Déu, Esplugues de Llobregat, Barcelona University, Member of the International Alternating Hemiplegia in Childhood Research Consortium IAHCRC and of the European Reference Network ERN EpiCARE, Barcelona, Spain; Department of Neurology (A.B., C.M.), Wake Forest School of Medicine, Winston-Salem, NC; Neurology Department (R.S.), Centro Hospitalar e Universitario do Porto–Hospital de Santo António, Porto, Portugal; Clinic for Child Neurology and Psychiatry (V.B., A.P.), Department of Child Neurology, Medical Faculty University of Belgrade, Serbia; Department of Human Genetics (Q.S.P.), Graduate School of Public Health, University of Pittsburgh, PA; Department of Pediatric Neurology (J.P.), Medical University of Silesia, Katowice, Poland; Clinical Neurosciences (K.V., J.H.C.), Developmental Neuroscience Programme, UCL Great Ormond Street Institute of Child Health, and Great Ormond Street Hospital for Children NHS Foundation Trust, Member of the International Alternating Hemiplegia in Childhood Research Consortium IAHCRC and of the European Reference Network ERN EpiCARE, London, UK; Sydney Children's Hospital (A.M.E.B.), Randwick; Department of Cardiology (A.M.D.), The Royal Children's Hospital, Melbourne, University of Melbourne; Department of Neurology (M.M.R.), Royal Children's Hospital, Melbourne; Agnes Ginges Centre for Molecular Cardiology (C.S.), Centenary Institute, University of Sydney; Epilepsy Research Centre (G.H., I.E.S.), Department of Medicine, University of Melbourne, Austin Health, Heidelberg, VIC; Department of Paediatrics (I.E.S.), University of Melbourne, Royal Children's Hospital, Florey and Murdoch Children's Research Institutes, Melbourne, Australia; Department of Clinical Epileptology, Sleep Disorders and Functional Neurology in Children (A.A., E.P.), University Hospitals of Lyon (HCL), Member of the International Alternating Hemiplegia in Childhood Research Consortium IAHCRC and of the European Reference Network ERN EpiCARE, Lyon, France; Paediatric Neurology Unit (I.C.), CMIN, Centro Hospitalar e Universitario Porto, Porto, Portugal; Clinical Neurophysiology Unit (C.Z.), IRCCS “E. Medea,” Bosisio Parini (LC), Italy; Department of Neurology (J.N.), CHUV and Université de Lausanne, Switzerland; Second Department of Neurology (K.D.), Institute Psychiatry and Neurology, Warsaw, Poland; Association AHC18+ e. V. (Germany) and Polish Association for People Affected by AHC, ahc-pl (M.P.); Department of Developmental Neurology (M.M.B.), Medical University of Gdańsk, Poland; Neurology Department (S.W.), University Hospital Antwerp; Neurogenetics Group (S.W.), University Antwerp, Belgium; First Department of Pediatrics (R.P.), “Agia Sofia” Children Hospital, National & Kapodistrian University of Athens, Greece; Department of Neurology (S.G.), University Medical Center of the Johannes Gutenberg University Mainz, Germany; Ion Channel Research Unit (D.S.S.), Department of Medicine/Cardiology and Pharmacology, Duke University Medical Center, Durham, NC; Cardiovascular Research Institute (G.S.P.), Weill Cornell Medical College, New York, NY; The Heart Centre (A.T.), Queen Mary University of London; Department of Pathology (M.A.), Great Ormond Street Hospital for Children NHS Foundation Trust; Department of Neuropathology (Z.M., M.T.), Institute of Neurology, University College London, UK; and ICT and Data Analysis Section (R.V.), Euro-Mediterranean Institute of Science and Technology (I.E.ME.S.T.), Palermo, Italy.

## Abstract

**Objective:**

To define the risks and consequences of cardiac abnormalities in *ATP1A3*-related syndromes.

**Methods:**

Patients meeting clinical diagnostic criteria for rapid-onset dystonia-parkinsonism (RDP), alternating hemiplegia of childhood (AHC), and cerebellar ataxia, areflexia, pes cavus, optic atrophy, and sensorineural hearing loss (CAPOS) with *ATP1A3* genetic analysis and at least 1 cardiac assessment were included. We evaluated the cardiac phenotype in an *Atp1a3* knock-in mouse (Mashl^+/−^) to determine the sequence of events in seizure-related cardiac death.

**Results:**

Ninety-eight patients with AHC, 9 with RDP, and 3 with CAPOS (63 female, mean age 17 years) were included. Resting ECG abnormalities were found in 52 of 87 (60%) with AHC, 2 of 3 (67%) with CAPOS, and 6 of 9 (67%) with RDP. Serial ECGs showed dynamic changes in 10 of 18 patients with AHC. The first Holter ECG was abnormal in 24 of 65 (37%) cases with AHC and RDP with either repolarization or conduction abnormalities. Echocardiography was normal. Cardiac intervention was required in 3 of 98 (≈3%) patients with AHC. In the mouse model, resting ECGs showed intracardiac conduction delay; during induced seizures, heart block or complete sinus arrest led to death.

**Conclusions:**

We found increased prevalence of ECG dynamic abnormalities in all *ATP1A3*-related syndromes, with a risk of life-threatening cardiac rhythm abnormalities equivalent to that in established cardiac channelopathies (≈3%). Sudden cardiac death due to conduction abnormality emerged as a seizure-related outcome in murine *Atp1a3*-related disease. *ATP1A3*-related syndromes are cardiac diseases and neurologic diseases. We provide guidance to identify patients potentially at higher risk of sudden cardiac death who may benefit from insertion of a pacemaker or implantable cardioverter-defibrillator.

The *ATP1A3* gene encodes the α-3 catalytic subunit of the neuronal ouabain-sensitive Na/K-ATPase complex. Na+/K + -ATPases are membrane-bound transporters regulating Na+ and K+ gradients through active ATP-dependent transport.^1^

The *ATP1A3*-related disorders are clinically heterogeneous and include a spectrum of at least 3 distinct, although overlapping, phenotypes: rapid-onset dystonia-parkinsonism (RDP)^2^; alternating hemiplegia of childhood (AHC)^1^; and cerebellar ataxia, areflexia, pes cavus, optic atrophy, and sensorineural hearing loss (CAPOS).^3^ Although rare, these conditions are important because they are generally severe, including paroxysmal events and chronic severely disabling neurologic deficits,^4^ with an increased rate of premature mortality. Epilepsy is often present, mostly in AHC; sudden death, including death during seizures or status epilepticus and apparent sudden unexpected death in epilepsy (SUDEP), is increasingly reported,^5,6^ but in fact the cause of death is usually unexplained. Newer phenotypes are emerging,^7,8^ suggesting that there may be additional unsuspected cases, perhaps also in sudden death cohorts.

In AHC, we previously demonstrated resting ECG abnormalities resembling those seen in inherited cardiac channelopathies, most commonly dynamic alteration of the repolarization phase.^9^ Additional published data in mice and humans suggest cardiac dysfunction in early death in AHC.^6,10–13^ No previous studies have investigated the cardiac phenotype of CAPOS or RDP.

We sought to determine whether the suggestions of cardiac involvement in *ATP1A3*-related disease had manifest clinical consequences beyond an ECG phenotype alone, noting that the single patient requiring intervention reported before^10^ might have had a coincidental comorbidity. The findings provide the basis for recommendations for clinical cardiac investigations and interventions in *ATP1A3*-related disease.

## Methods

### Patients

**Figure 1:**
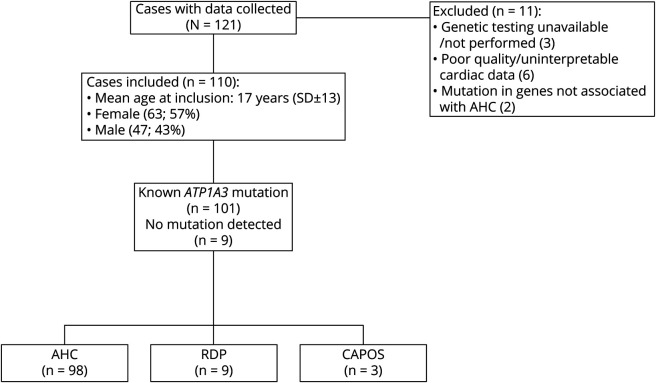
Study design Diagram illustrating study design with included and excluded cases. AHC = alternating hemiplegia of childhood; CAPOS = cerebellar ataxia, areflexia, pes cavus, optic atrophy, and sensorineural hearing loss; RDP = rapid-onset dystonia-parkinsonism.

Patients were recruited from 19 participating centers in 13 different countries: Duke University Medical Center, Durham, NC (n = 21); Istituto Giannina Gaslini, University of Genoa, Italy (n = 17); Hospital Sant Joan de De'u Barcelona, Spain (n = 13); Wake Forest School of Medicine, Winston-Salem, NC (n = 11); The National Hospital for Neurology and Neurosurgery, UK (n = 8); Department of Pediatric Neurology, Medical University of Silesia, Katowice, Poland (n = 6); University Clinic for Child Neurology and Psychiatry, Belgrade, Serbia (n = 5); Great Ormond Street Hospital for Children, UK (n = 5); Royal Children's Hospital, Melbourne, Australia (n = 4); C. Besta Neurologic Institute Milan, Italy (n = 4); Department of Clinical Epileptology, Sleep Disorders and Functional Neurology in Children, University Hospitals of Lyon, France (n = 2); Neuropediatric Department, Hospital Maria Pia do Centro Hospitalar do Porto, Portugal (n = 2); IRCCS E. Medea, Italy (n = 2); Centre Hospitalier Universitaire Vaudois, Lausanne, Switzerland (n = 2); Department of Neurology, Institute of Psychiatry and Neurology, Warsaw, Poland (n = 2); Department of Developmental Neurology, Medical University of Gdansk, Poland (n = 2); Department of Neurology, University Hospital Antwerp, Belgium (n = 2); Hospital “Agia Sofia,” Athens, Greece (n = 1); and University Medical Center of the Johannes Gutenberg University Mainz, Germany (n = 1). Patients meeting the clinical diagnostic criteria for typical AHC or other *ATP1A3*-associated syndromes and who underwent genetic analysis of *ATP1A3*, with or without identified mutations in *ATP1A3*, were included. At least 1 cardiac evaluation was required from the following: ECG, echocardiogram and prolonged ECG (≥24 hours), or prolonged ECG recording from EEG-videotelemetry. For patients who had already been included in the first study,^9^ at least 1 further cardiac investigation was required. Eleven patients were excluded due to genetic testing being unavailable or not performed (n = 3), poor-quality/uninterpretable cardiac data (n = 6), or pathogenic mutation in genes not associated with AHC being identified (n = 2) (figure 1). Recruitment and data collection were from September 1, 2015, until September 1, 2018. Clinical and genetic data were collected through a standardized questionnaire. *ATP1A3* mutations were identified by Sanger, whole-exome, or whole-genome sequencing. De novo mutation status was evaluated by Sanger sequencing when parental DNA was available; when unavailable, pathogenicity was declared if the same mutation was previously reported as de novo in another patient with an *ATP1A3*-related condition.

A total of 110 cases were recruited, including 98 with AHC, 9 with RDP, and 3 with CAPOS. All cases had identified mutations in *ATP1A3* except for 9 cases of AHC with no mutation in *ATP1A3* detected by direct gene sequencing (these were included because they all met the clinical diagnostic criteria for AHC^14^) and no other causative mutations identified on exome sequencing or gene panels. Of the 98 cases of AHC, 22 had been reported in the previous study.^9^ None of the cases of RDP or CAPOS were previously reported.

We calculated a severity index for the patients with AHC that was based on clinical features previously associated with a gradient of clinical severity in a relatively large cohort of patients with AHC.^15^ A score of 1 was assigned for each of the following clinical variables: early onset of paroxysmal episodes (≤2 months), tonic/dystonic attacks, plegic attacks, seizures/epilepsy, status epilepticus, episodes of autonomic dysfunction, gait unsteadiness or ataxia, dystonia, speech or language disorders including dysarthria, and intellectual disability. We evaluated the correlation of this cumulative severity index obtained for each patient with AHC with the presence of ECG abnormalities and dynamic abnormalities.

### Cardiac investigations

Original cardiac investigations were anonymized, scanned, collected, and reviewed centrally by 2 independent cardiologists with expertise in genetic cardiac disease and sudden cardiac death (R.A.G-R, J.P.K.). Abnormal repolarization was defined by the presence of abnormal T-wave morphology (flattened or biphasic T waves; bifid or notched T waves) or T-wave inversion in 2 or more of the following leads: I, aVL, and V_4_ through V_6_ (lateral repolarization abnormalities); II, III, and aVF (inferior repolarization abnormalities); and V_1_ through V_3_ in patients ≥14 years of age (anterior repolarization abnormalities); repolarization abnormalities of this type are seen in 2% of healthy adults.^16^ Widespread repolarization abnormalities were defined by abnormalities present in >1 group of leads. The QTc interval was calculated from lead II with the Bazett formula^17^; its normal range is 360 to 460 milliseconds.^18^ The Brugada pattern is characterized by a coved-type ST-segment elevation ≥2 mm followed by a negative T wave in ≥1 of the right precordial leads V_1_ to V_2_. Early repolarization (ER) was defined as a deflection in the R-wave descent (slurred pattern) or a positive deflection with a secondary r′' wave (notching pattern) in the terminal part of the QRS complex in at least 2 of the following leads: I, avL, and V_4_ through V_6_ (lateral ER) or II, III, and avF (inferior ER).^19^ Lateral ER is seen in 3.5% of healthy individuals, and inferior ER appears in 2.4%.^20^ The U wave is defined as a small upward deflection following the T wave. It is discernible in ≈25% of the healthy population when the heart rate is within 80 to 95 bpm and is not detectable when the heart rate is >95 bpm.^21^ Right bundle-branch block, complete (RBBB) and incomplete (IRBBB), and intraventricular conduction delays (IVCDs) were defined according to established criteria.^22^ Isolated IVCD was considered normal in the absence of additional ECG abnormalities because it is seen in up to 5% of the normal population.^23,24^ Isolated RBBB is seen in 2% to 4% of healthy individuals.^24^

### Pathology

The ATP1A3 subunit is expressed in the human heart and in neurons of the cerebral cortex and other brain structures,^25^ with the highest expression detected in the frontal cortex.^26^ Immunolabeling for ATP1A3 was performed in adult human heart from a 75-year-old man who died of bronchopneumonia after postmortem examination.

### Mouse model

Generation of the mouse model was performed as described before.^12^ The aims of the *ATP1A3* D801N knock-in mouse (Mashl^+/−^) model study were to establish the presence of baseline ECG abnormalities in this model compared to wild-type (WT) mice and to determine the sequence of events in cardiac death resulting from seizure activity.

#### Experiment 1

The interictal EEGs of 2 groups of mice were recorded and compared. Group 1 consisted of adult WT mice; group 2 consisted of age-matched D801N (Mashl^+/−^) mice of C57BL background. ECGs were obtained as described previously.^27–29^

#### Experiment 2

ECGs were recorded in Mashl^+/−^ mice during seizures induced by intra-amygdalar injection of kainic acid, ending in death, to determine the type of arrhythmias that may be associated with seizure-associated cardiac arrest and death. Mice underwent surgical implantations to record intracranial EEG and ECG after kainic acid injection.

#### ECG analysis

All ECG data were saved in 2-second frame shots and analyzed with ImageJ software. The PR interval was calculated by horizontal measurement between the peak of the P wave to the peak of the R wave, QRS interval from the Q peak to the S peak, RR from 2 consecutive R waves, and QT from the Q peak to the end of the T wave. The end of the T wave was defined using a tangent method in which a line was drawn representing the isoelectric line, which is defined by the base of the P wave to the base of the T wave, and a tangent drawn along the steepest slope of the T-wave repolarization. The end of the T wave is defined as the cross between the tangent and the isoelectric line. The QT interval was corrected with the Mitchell equation modified for mouse physiology.^28^

### Statistical analysis

Data were tested for normal distribution. The significance of differences in clinical and genetic factors potentially associated with ECG changes was estimated by Pearson χ^2^ or 2-sided Fisher exact test, as appropriate, for categorical variables and by Wilcoxon rank-sum test for continuous variables. Missing data were <5% of the total number of cases for all analyzed variables; they were omitted from analysis. *p* Values were considered significant at 0.05 (adjusted due to multiple testing with the Bonferroni method). Data were analyzed with Stata/IC 11.1(StataCorp, College Station, TX).

### Standard protocol approvals, registrations and patient consents

This research was approved by local review boards or ethics committees. For all cases, written informed consent for research use of clinical and genetic data was obtained from patients, their parents, or legal guardians in the case of minors or those with intellectual disability. All animal procedures were approved by the Duke University Institutional Animal Care and Use Committee and were conducted in accordance with the US Public Health Service's Policy on Humane Care and Use of Laboratory Animals.

### Data availability

The authors confirm that the data supporting the findings of this study are available from the corresponding authors on reasonable request.

## Results

### Demographic and clinical features

One hundred ten patients (63 female, 47 male) were included in the analysis. Mean age at inclusion was 17 years (SD ± 13, range 1–64 years), with 64 patients <17 years of age. A history of seizures or epilepsy was reported in 58 patients (53%), with status epilepticus in 21. Episodes of autonomic dysfunction, including breathing difficulties, were reported in 60 patients (55%). The only reported symptom considered to be related to cardiac function was syncope, reported in 3 patients (3%).

### Cardiac investigations

Investigations included 12-lead ECGs in 99 patients (≥2 in 18 patients), Holter ECG in 65 patients (serial in 2 patients), echocardiogram in 80 patients, and an ajmaline provocation test in 1 patient.

The first 12-lead ECG available was performed at an average age of 18 years (SD ± 15 years) and showed abnormalities in 60 of 99 patients (61%). The most common changes were T-wave abnormalities anteriorly (32%), laterally (23%), and inferiorly (42%). Thirteen patients (13%) had widespread repolarization abnormalities. Lateral ER was seen in 5 of 99 patients; inferior ER was seen in 8. In 3 of 99 patients, there was ≥2-mm anterior J-point elevation, but no Brugada pattern was observed on the 12-lead ECG. The average QTc was 382 milliseconds (median 375 milliseconds). Thirty-one (31%) patients had a QTc ≤360 milliseconds. Only 1 patient had a prolonged QTc interval (500 milliseconds) and was not on any drug treatment at the time of the ECG. U waves were present in 16 patients (16%), and in 2 of them, the heart rate was >95 bpm. Other abnormalities included left axis deviation (6%), right axis deviation (8%), IVCD (21%), and IRBBB (23%). The ECG was repeated in 18 patients ≥1 times (average interval time 2 years, SD ± 2 years) and showed dynamic changes in 10 of 18 (56%). Details of the 12-lead ECGs are provided in the table.

**Table:**
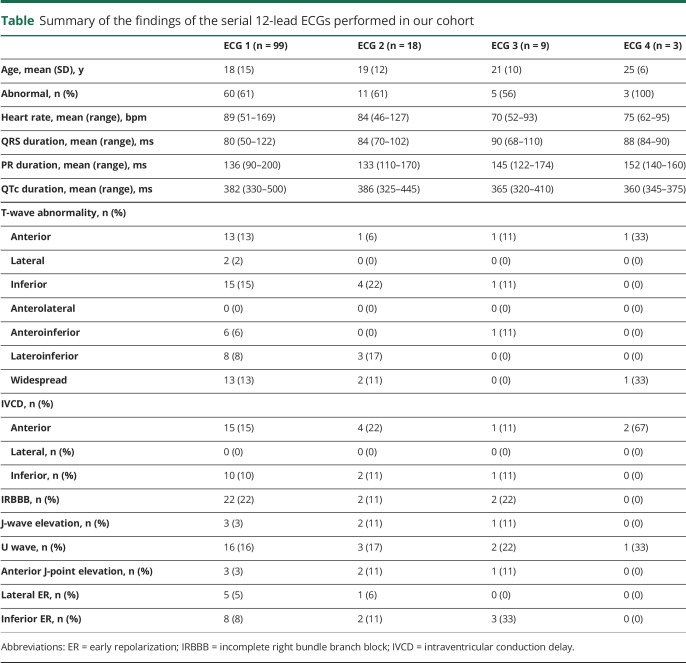
Summary of the findings of the serial 12-lead ECGs performed in our cohort

**Figure 2:**
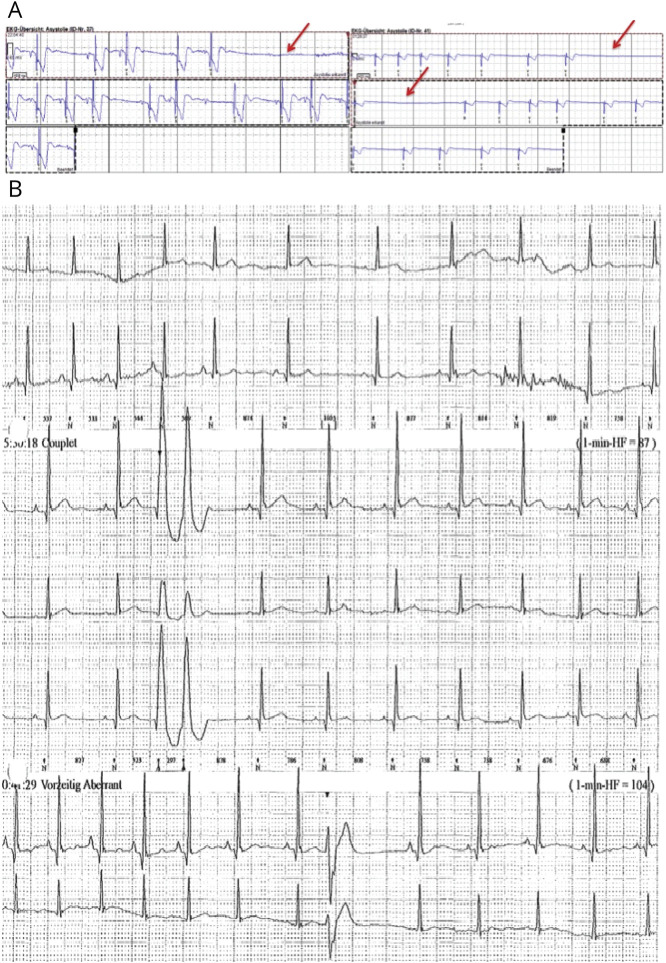
Holter abnormalities in a young patient with AHC requiring the insertion of an ICD (A) Abnormal Holter ECG showing asymptomatic sinus pauses of up to 4 seconds in duration (red arrows) and (B) polymorphic ventricular ectopics in couplets and bigeminy in a young patient who required insertion of an implantable cardioverter-defibrillator (ICD) at the age of 27 years (patient with alternating hemiplegia of childhood [AHC], c.2401G>A p.D801N mutation).

The first Holter ECG was performed at an average age of 17 years (SD ± 13 years) and showed abnormalities in 24 patients (24 of 65, 37%), mainly T-wave abnormalities (n = 20), including dynamic T-wave inversion (n = 2) or T-wave notching (n = 9), and dynamic J point elevation (n = 9). We documented significant QT prolongation in 2 patients (505 and 480 milliseconds, respectively) not exposed to QT-prolonging drugs and QT shortening in 1 patient (330 milliseconds). Isolated supraventricular ectopics were present in 34 patients (34 of 65, 52%), and ventricular ectopics were seen in 23 (23 of 65, 35%); 4 patients had frequent ventricular ectopics, including couplets and bigeminy. Three patients (3 of 65, 5%) had evidence of conduction abnormalities on ambulatory Holter monitoring, in the form of pathologic sinus pauses in all 3, with additional atrioventricular block in 1 patient. Two patients had repeated Holter ECGs. One patient had 2 Holter ECGs, both showing no arrhythmias despite widespread repolarization abnormalities and IRBBB on the resting 12-lead ECG. The second patient was a 28 year-old woman with recurrent repolarization abnormalities on 12-lead ECGs; she had 5 repeat Holter ECGs. These showed sinus pauses, atrioventricular block, and atrial and ventricular ectopics (figure 2); she subsequently underwent insertion of an implantable cardioverter-defibrillator (ICD).

Overall, there were resting or ambulatory ECG abnormalities in 71 patients (71 of 110, 65%). A sensitivity analysis was conducted by excluding the 22 patients included in the previous study^9^: ECG (either 12-lead or Holter) abnormalities were present in 55 patients (55 of 88, 63%), and dynamic changes in serial 12-lead ECGs were present in 6 patients (6 of 13, 46%).

Echocardiography was performed at an average age of 16 years (SD 12 years) and did not show evidence of structural heart disease in any case. Only 1 patient with AHC carrying the *ATP1A3* mutation c.2839G>C-G947R, who was 46 years of age, had left ventricular hypertrophy. This was likely associated with previously undiagnosed essential hypertension detected 17 months after the echocardiogram and for which he is currently on treatment with lisinopril.

One patient with AHC who had previously been found to have features suggestive of Brugada syndrome (mild prolongation of QRS and J-point elevation) on a single-lead ECG (modified V_1_) during EEG-videotelemetry recording^9^ underwent ajmaline provocation testing, which revealed an RSR′ pattern to the QRS complex with subtle J-point elevation in lead V_2_ placed in the second intercostal space (high parasternal position) but did not meet the diagnostic criteria for Brugada syndrome.^30^ Previous and subsequent 12-lead ECGs were unremarkable, confirming the dynamic nature of the abnormalities.

### Patients requiring intervention

Three patients underwent implantation of a loop recorder and, on the basis of the findings recorded, had subsequent implantation of a permanent pacemaker or ICD. None of them were on pharmacologic treatment that could adversely affect the cardiac conduction system.

A female patient with AHC (c.2401G>A; p.D801N mutation in *ATP1A3*) had syncopal episodes, in addition to hemiplegic attacks and epileptic seizures. She was included in the study at 26 years of age. Her 12-lead ECGs showed dynamic changes, with repolarization abnormalities including lateral ER, inverted T waves in the anterior leads, and U waves. She also had consistently short QTc intervals (320–milliseconds). She had 5 repeat Holter ECGs and a loop recorder implanted that showed multiple asymptomatic pauses (up to 4.4 seconds), paroxysmal complete atrioventricular block, atrial ectopics and polymorphic ventricular ectopics in couplets, and bigeminy (figure 2). Her echocardiogram showed a structurally and functionally normal heart. Aged 27 years of age, an ICD was implanted due to the combination of syncope, atrioventricular block, and ventricular ectopy. Only 1 episode of “collapse” has occurred since (during 19 months of follow-up), but no rhythm disturbances were seen on ICD interrogation during the episode.

A female patient with AHC (c.410C>T; p.S137F mutation in *ATP1A3*) started experiencing episodes of loss of consciousness with respiratory arrest at 21 years of age^10^ Her routine 12-lead ECG was normal. She underwent implantation of a cardiac loop recorder at 23 years of age, which documented 3 episodes of asystole >3 seconds over a period of 4 months; a cardiac pacemaker was implanted. She had had EEG-videotelemetry before pacemaker implantation. She did not experience any episodes of loss of consciousness with respiratory arrest during the recording. The single-lead ECG that was part of the telemetry showed no abnormalities. She has remained free of episodes of loss of consciousness with respiratory arrest after the pacemaker insertion, and there has been no evidence of ventricular arrhythmia on pacemaker interrogation over 7 years of follow-up.

A female patient with AHC (negative for *ATP1A3* mutation) had an onset of hemiplegic attacks at 6 months of age and onset of syncopal episodes at ≈1 year of age. Bradycardia was also noted during the fetal period. Her 12-lead ECGs showed dynamic changes with intermittent junctional rhythm, low QRS voltages, delayed RS progression, supraventricular ectopics, and widespread repolarization abnormalities. Holter ECG was abnormal with supraventricular and ventricular ectopy. An echocardiogram showed left ventricular hypertrabeculation, not fulfilling diagnostic criteria for left ventricular noncompaction, with preserved systolic function and was otherwise unremarkable. Due to the ongoing syncopal episodes, she had a loop recorder inserted at 2 years of age, which revealed sinus pauses >4 seconds. A pacemaker was subsequently inserted with no recurrence of the syncopal episodes over 6 years of follow-up.

### Analysis of potential clinical and genetic risk factors

The prevalence of repolarization abnormalities on 12-lead ECGs was greater in patients ≥16 years of age (36 of 48, 75%) than in those <16 years (24 of 51, 47%) (*p* = 0.01). In contrast, the prevalence of repolarization and conduction abnormalities on Holter ECGs was greater in the younger patients (16 of 30, 53%) than in the older patients (8 of 35, 23%) (*p* = 0.01). There was no difference in the prevalence of ECG abnormalities by sex or in the prevalence of dynamic changes by age or sex.

Abnormalities on the 12-lead ECG were found in all 3 *ATP1A3*-related disease categories with no difference in prevalence (*p* > 0.99): 52 of 87 (60%) patients with AHC, 2 of 3 (67%) patients with CAPOS, and 6 of 9 (67%) patients with RDP. Serial ECGs were available in 18 patients with AHC, with 10 (56%) showing dynamic changes, while in 2 patients with CAPOS, there were no dynamic changes (1 had consistently normal ECG, 1 had repeatedly abnormal ECG).

Abnormalities on the Holter ECG were also found in all the categories except CAPOS, for which Holter ECGs were performed in 2 patients and were unremarkable. Otherwise, Holter ECGs were abnormal with evidence of conduction disease in patients with AHC only (4 of 61, 7%), and repolarization abnormalities were seen in 19 patients with AHC (19 of 61, 31%) and 1 patient with RDP (1 of 2, 50%).

In 9 of the 98 patients with AHC, no mutation in the *ATP1A3* gene was identified; 3 of them had serial ECGs, and 2 of the 3 presented dynamic changes. Abnormalities on the 12-lead ECG were present in 6 of 9 (67%) and abnormalities on Holter monitoring were seen in 5 of 9 (56%) of the *ATP1A3*-negative patients. These were the same type of abnormalities observed in the *ATP1A3* mutation carriers. There was no difference in the prevalence of ECG abnormalities in patients with AHC with or without *ATP1A3* mutation (*p* > 0.99).

**Figure 3:**
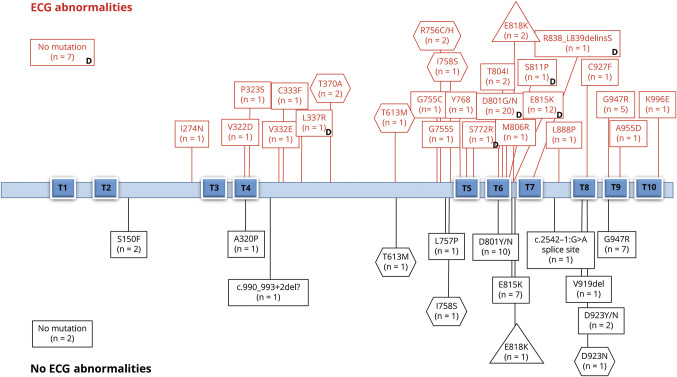
ECG abnormalities in *ATP1A3*-related syndromes Graphic representation of the *ATP1A3*-related syndromes (alternating hemiplegia of childhood [AHC] = rectangles, cerebellar ataxia, areflexia, pes cavus, optic atrophy, and sensorineural hearing loss [CAPOS] = triangles, rapid-onset dystonia-parkinsonism [RPD] = hexagons) (n = 110), associated mutations, and prevalence of ECG (12-lead and/or Holter) abnormalities. Each box includes the number of cases with a specific mutation and ECG abnormalities in the upper part or without ECG abnormalities in the lower part. Reference sequences for the corresponding ATP1A3 transcript and protein were NM_152296.4 and Uniprot P13637, respectively. T1 through T10 are transmembrane domains. The position of the mutation across the functional domains was not associated with different prevalence of ECG abnormalities or dynamic changes. In 9 patients with AHC, no mutation was identified in *ATP1A3*. D = dynamic changes (when serial tests were available).

The position of the mutation across the functional domains was not associated with the different prevalence of ECG abnormalities or dynamic changes. Details of the different syndromes, mutations, and abnormalities are presented in figure 3.

Of 110 patients, 77 (70%) were exposed to treatment with a potential effect on cardiac conduction at the time of the investigation, the most frequent being flunarizine,^31^ topiramate,^32^ and benzodiazepines.^33^ There was no difference in the prevalence of ECG abnormalities (*p* > 0.99) or dynamic changes (*p* > 0.99) between patients exposed and those not exposed to treatment with potential effect on cardiac conduction or repolarization.

The average severity clinical severity index in the AHC cohort was 7 (range 1–10). No significant correlation emerged between phenotypic severity and prevalence of ECG abnormalities or dynamic abnormalities.

### Pathology

**Figure 4:**
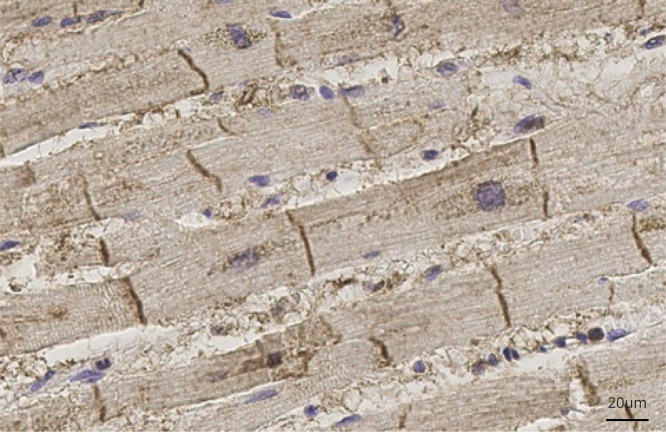
ATP1A3 expression in an adult normal heart Dark brown stripes show strong immunolabeling for ATP1A3 corresponding to intercalated disks in adult myocardium from a 75-year-old man (cause of death at postmortem: bronchopneumonia). Tissue samples were fixed in formalin and embedded in paraffin. A standard immunohistochemistry method was applied to 5-μm-thick sections with primary antibody anti-ATP1A3 (Santa Cruz, polyclonal, goat, sc16052) at a dilution of 1:1,000 with overnight incubation at 4°C in diluent buffer (DAKO REAL, Ab diluent S2022). Immunostaining was qualitatively evaluated.

Immunolabeling for ATP1A3 was performed in an adult human heart from a 75-year-old man who died of bronchopneumonia. As shown in figure 4, this confirmed strong expression of ATP1A3 in human cardiomyocytes.

### Mouse model

#### Experiment 1

**Figure 5:**
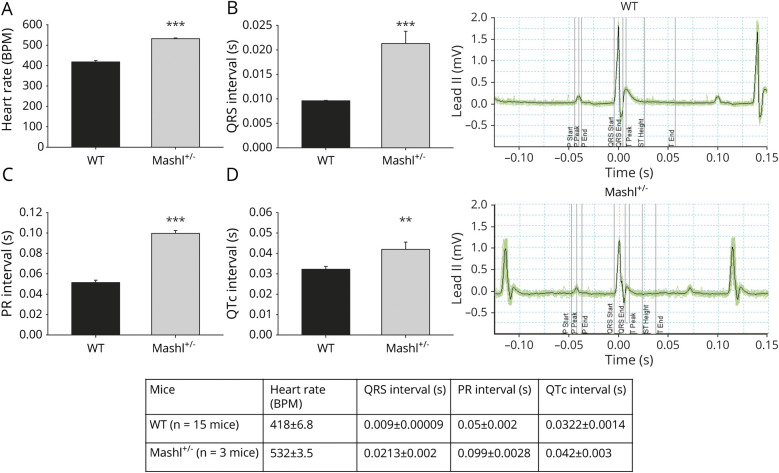
ECG data in the Mashl+/− compared to WT mice Comparison of ECG data acquired from wild-type (WT; n = 15) and Mashl^+/−^ mice (n = 3). (A) Heart rate, (B) QRS interval, (C) PR interval, and (D) QTc interval. Traces are examples of ECG traces in WT and mutant mice. Heart rate, QRS, and PR interval were higher in Mashl^+/−^ mice. ***p ≤ 0.001, **p < 0.01 (Student *t* test). BPM = beats per minute.

At baseline, Mashl^+/−^ mice had increased heart rate (532 ± 3.5 vs 418 ± 6.8 beats per minute, *p* < 0.001), prolonged QRS (0.0213 ± 0.002 vs 0.009 ± 0.00009 seconds, *p* < 0.001), prolonged PR interval (0.099 ± 0.0028 vs 0.05 ± 0.002 seconds, *p* < 0.001), and longer QTc interval (0.042 ± 0.003 vs 0.0322 ± 0.0014 seconds, *p* < 0.01) compared to WT littermates (WT n = 15, Mashl^+/−^ n = 3) (figure 5).

#### Experiment 2

**Figure 6:**
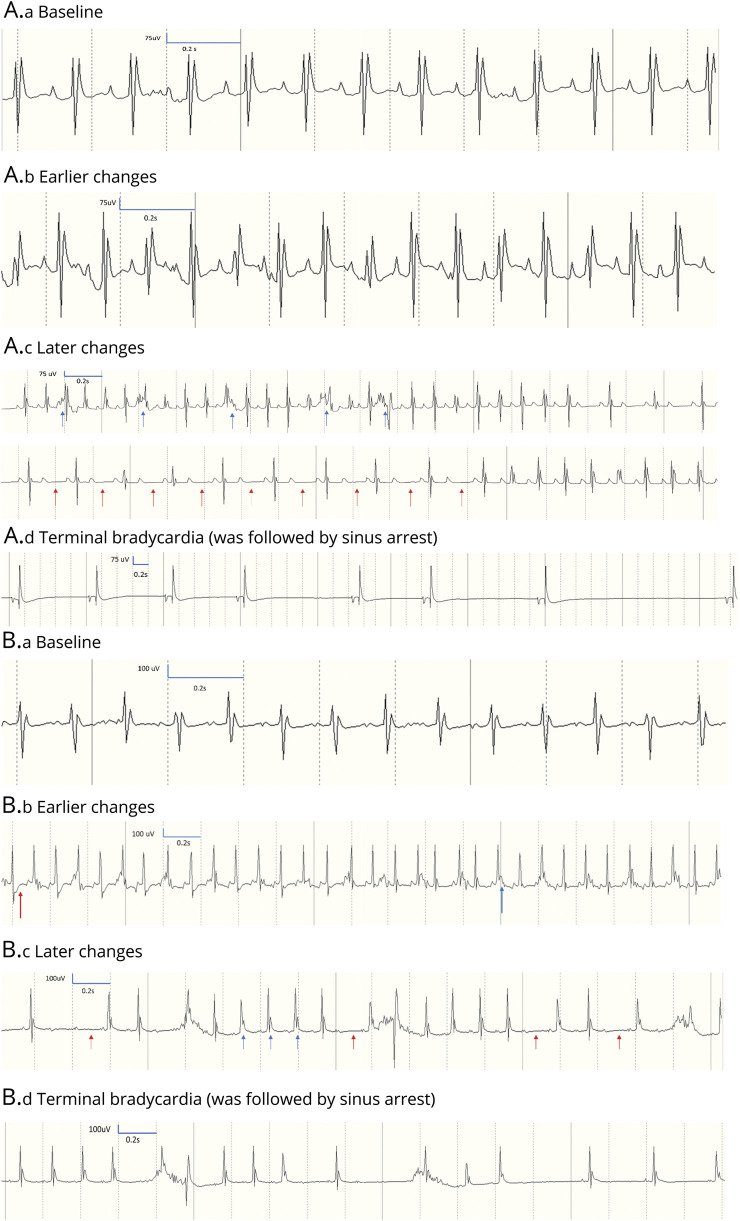
ECG abnormalities in the Mashl+/− mice (n = 3) after seizure induction (A) Mashl+/− No. 1 ECG traces. (A.a) Baseline, with normal heart rate, noise present stems from skeletal muscle (breathing) activity. (A.b) Earlier changes started with onset of EEG seizures at 21 minutes after injection: heart rate increase and JT-segment elevation. (A.c) Later changes. Consecutive ECG traces shortly before sinus arrest showing heart rate fluctuation and atrioventricular block (red arrows); premature ventricular contractions are also visible (blue arrows). (A.d) Terminal change. Sinus bradycardia that was followed by sinus arrest. (B) Mashl^+/−^ No. 2 ECG traces. (B.a) Baseline, with normal heart rate. (B.b) Earlier changes started with onset of EEG seizures after 5 minutes of injection and persisted to 1 minute before death (time of B.b. illustration). Increased heart rate with JT-segment depression (red), and JT elevation (blue) widened QRS. (B.c) Later changes. Sequence of events <1 minute before sinus arrest showing atrioventricular block (red arrows) and elevated JT segments (blue arrows). (B.d) Terminal change. Sinus bradycardia that was followed by sinus arrest. Mashl^+/−^ No. 3 had similar ECG traces from baseline to terminal changes.

After injection of kainic acid, all mice showed elevated JT intervals in response to ictal activity. In addition, 1 of the 3 Mashl^+/−^ mice showed a period with JT-segment depression (figure 6A). Two Mashl^+/−^ mice died of atrioventricular block (figure 6B).

## Discussion

We show that *ATP1A3*-related diseases can cause heart abnormalities and neurologic manifestations, with a requirement for lifesaving cardiac intervention equivalent to that for the better-known genetic cardiac channelopathies.^18^ Nearly 3% (3 of 98) of patients with AHC showed asymptomatic and symptomatic asystole (and ventricular arrhythmias) and required the insertion of a pacemaker or ICD. Our previous observation in 1 *ATP1A3*-related condition of a significantly increased prevalence of abnormalities of the resting ECG, including abnormalities of repolarization reminiscent of genetic cardiac channelopathies, compared with healthy controls and disease controls with epilepsy,^9^ was replicated and extended here. We found ECG abnormalities in 65% of patients, with dynamic changes in the 56% of patients who had serial ECGs, across all *ATP1A3*-related syndromes and some patients with AHC with no identified mutation in *ATP1A3*. The location of the mutation and the exposure to treatment with potential effects on cardiac conduction were not associated with different prevalence of ECG abnormalities or dynamic changes.

There are some limitations that need to be acknowledged. The study is unpowered to interpret the results in patients without AHC due to the small sample size of the non-AHC cohorts. Data collection was partly retrospective. Twenty-two cases with AHC had already been included in the previous study,^9^ therefore introducing a potential selection bias. To minimize this bias, we have conducted a sensitivity analysis that confirmed the high prevalence of resting and/or ambulatory ECG abnormalities and dynamic changes.

Nine patients with an AHC phenotype did not have a mutation in *ATP1A3* on direct gene sequencing, but the majority (6 of 9, 67%) had the same dynamic cardiac electrical abnormalities observed in the *ATP1A3* mutation carriers, with 1 patient even requiring the insertion of a pacemaker due to symptomatic conduction disease. This suggests that mutations might have been missed, a recognized phenomenon, that there may be mosaicism, or that mutations in a different gene might be causative (suggesting cardiac dysfunction is related to shared pathophysiology rather than to *ATP1A3* mutations only).

The underlying basis of the ECG abnormalities observed is not yet explained but, as suggested in our previous study, may be related to dynamic abnormality of cardiac repolarization reserve. In the present study, we confirmed the expression of ATP1A3 in the structurally and histologically normal heart of an adult man who died of bronchopneumonia and had postmortem examination. The presence of dynamic ECG changes, the frequency of necessary preventive intervention (i.e., pacemaker or ICD), and the apparent age-related penetrance underpin similarities to the genetic cardiac channelopathies. Although there was a lower prevalence of 12-lead ECG abnormalities in patients <16 years of age, Holter monitoring was more frequently abnormal in younger patients, suggesting less manifest and more dynamic cardiac electric abnormalities at younger ages. This is in keeping with findings in cardiac sodium channel loss-of-function mutations, in which presentation with more severe conduction disease and atrial arrhythmia is commoner in childhood and adolescence, whereas a Brugada phenotype is commoner in adulthood,^34^ suggesting an age-related penetrance to the disease. Although the cardiac abnormalities demonstrated in this study are not due to structural abnormalities detectable on echocardiography, it is theoretically possible that there may be structural abnormalities too subtle to be observed on echocardiography; additional investigations such as cardiac MRI with gadolinium contrast or histology from biopsies or postmortem heart tissue may provide further information.

The asystolic episodes recorded in our 3 patients occurred independently to, and showed a different semiology from, other paroxysmal events (e.g., hemiplegia or seizures). This is very important to consider in the clinical assessment of *ATP1A3*-related disorders, in which multiple types of paroxysmal episodes often coexist and there is increasing evidence of the risk of sudden death or progressive disease course.^4,6^ The episodes of sudden death reported in patients with AHC^6^ have usually been ascribed to SUDEP, but they may be directly caused by fatal cardiac arrhythmias. Cardiac causes of sudden death have different implications in terms of preventive strategies: achieving better seizure control can reduce the risk of SUDEP, while pacemaker or ICD insertion can be lifesaving for life-threatening conduction disease or cardiac arrhythmias.^35^

We evaluated the cardiac phenotype in Mashl^+/−^ mice, carrying the heterozygous D801N mutation, present in about one-third of patients with AHC.^1^ Resting ECGs in the mutation-carrying mice under general anesthesia revealed evidence of intracardiac conduction delay, with a prolonged PR interval, indicative of likely conduction delay in the atria and atrioventricular node. In addition, there was a prolonged QRS duration, revealing probable delayed conduction in the His-Purkinje system and ventricle. When challenged by induced seizures, the mice were initially in sinus rhythm, but with time, abnormalities emerged, with heart block or complete sinus arrest leading to death. In WT mice receiving kainic acid, preictal, but not ictal, tachycardia was observed, and the squared coefficient of variation of R-R intervals was significantly elevated before and during seizures compared to control conditions.^36^ In WT rats, kainic acid–induced seizures produced an immediate initial bradycardic response coinciding with initial low-level seizure activity followed by subsequent tachycardia with QTc prolongation and T-wave elevation with increasing seizure activity.^37^ Our observations of multiple cardiac rhythm abnormalities during kainic acid–induced seizures appear to be more remarkable than those described above in WT rodents in the literature.^36,37^ Our animal data showed resting ECG abnormalities similar to those observed in humans with AHC and other *ATP1A3*-related conditions. An important difference is that the murine conduction abnormalities deteriorate in the context of seizure activity, which so far has not been recorded in humans, but we note that the seizure paradigm in the experimental setting differs from most self-terminating seizures in humans. However, we cannot exclude that seizures represent a precipitating factor for cardiac arrhythmias in compromised tissue also in humans.

*Atp1a3* mutations may indirectly affect the heart rhythm in mutant mice through the excessive excitability of the brain and its predisposition to spreading depression,^11^ which has been shown to be a mechanism for autonomic dysfunction leading to SUDEP.^27^ A direct effect of the mutation on the heart is another potential mechanism: although Atp1a3 has not been detected in the heart of >2-month-old adult mice, it has been shown to be expressed in myocardium in mouse embryos at embryonic days 10.5 and 16.5,^38^ raising the possibility of a congenital effect of the mutated gene on cardiac electrophysiology. Further work is required and may help in the understanding of the cardiac pathophysiology in humans.

Our study offers an example of the precision medicine paradigm for rare conditions in which the underlying genetic etiology informs management and treatment strategies. Our findings provide further robust evidence that all patients with *ATP1A3*-related conditions should have longitudinal and systematic cardiac assessment by cardiologists with expertise in inherited cardiac disease. Our findings also provide evidence to recommend implantation of a loop recorder in all patients with *ATP1A3*-related disorders with atypical events (i.e., syncope or any other paroxysmal event with features atypical for seizures or hemiplegic attacks) to identify patients potentially at higher risk of sudden cardiac death who may benefit from insertion of pacemaker or ICD. Whether implantation of loop recorders in individuals with abnormal ECGs but no cardiac symptoms is warranted remains to be determined. We also recommend adoption of the current accepted practice for cardiac ion channel disease, which is also characterized by dynamic ECG changes.^18^ Baseline ECG, cardiac ultrasound, and Holter ECG should be undertaken in every patient with an *ATP1A3*-related condition, and then annual 12-lead ECG screening should be continued, with additional investigations (e.g., Holter monitoring) guided by symptoms or clinical status. Further prospective studies of cardiac disturbances in *ATP1A3*-related conditions to test the utility and cost-effectiveness of this approach and to identify potential precipitating factors for life-threatening cardiac arrhythmias are warranted.

## Glossary

AHC: alternating hemiplegia of childhood
CAPOS: cerebellar ataxia, areflexia, pes cavus, optic atrophy, and sensorineural hearing loss
ER: early repolarization
ICD: implantable cardioverter-defibrillator
IRBBB: incomplete RBBB
IVCD: intraventricular conduction delay
RBBB: right bundle-branch block
RDP: rapid-onset dystonia-parkinsonism
SUDEP: sudden unexpected death in epilepsy
WT: wild-type
